# Dendrimer Technology in Glioma: Functional Design and Potential Applications

**DOI:** 10.3390/cancers15041075

**Published:** 2023-02-08

**Authors:** Hallie Gaitsch, Andrew M. Hersh, Safwan Alomari, Betty M. Tyler

**Affiliations:** 1 Department of Neurosurgery, Johns Hopkins University School of Medicine, Baltimore, MD 21287, USA; 2 NIH Oxford-Cambridge Scholars Program, Wellcome—MRC Cambridge Stem Cell Institute and Department of Clinical Neurosciences, University of Cambridge, Cambridge CB2 1TN, UK

**Keywords:** dendrimers, drug delivery, gene therapy, glioblastoma, glioma, imaging, nanotechnology

## Abstract

**Simple Summary:**

Gliomas are common primary brain tumors that account for a high number of cancer-related deaths worldwide. Patients with aggressive gliomas have poor prognoses due to resistance to current treatment regimens. Anatomical barriers between the brain and the peripheral circulation make glioma drug delivery particularly challenging. Recently, the potential for dendrimers—nanomaterials characterized by a unique branching structure with many binding sites—to deliver therapeutic and diagnostic cargo directly to the site of gliomas has been explored. An increasing number of studies in glioma cell lines and animal models suggest that this dendrimer-based delivery approach may serve to decrease drug toxicity and allow for the use of nontraditional therapeutic and imaging agents in glioma. Here, we review the physical characteristics of dendrimers, strategies for modifying dendrimers to target glioma cells, and novel applications of this nanotechnology in glioma models.

**Abstract:**

Novel therapeutic and diagnostic methods are sorely needed for gliomas, which contribute yearly to hundreds of thousands of cancer deaths worldwide. Despite the outpouring of research efforts and funding aimed at improving clinical outcomes for patients with glioma, the prognosis for high-grade glioma, and especially glioblastoma, remains dire. One of the greatest obstacles to improving treatment efficacy and destroying cancer cells is the safe delivery of chemotherapeutic drugs and biologics to the tumor site at a high enough dose to be effective. Over the past few decades, a burst of research has leveraged nanotechnology to overcome this obstacle. There has been a renewed interest in adapting previously understudied dendrimer nanocarriers for this task. Dendrimers are small, highly modifiable, branched structures featuring binding sites for a variety of drugs and ligands. Recent studies have demonstrated the potential for dendrimers and dendrimer conjugates to effectively shuttle therapeutic cargo to the correct tumor location, permeate the tumor, and promote apoptosis of tumor cells while minimizing systemic toxicity and damage to surrounding healthy brain tissue. This review provides a primer on the properties of dendrimers; outlines the mechanisms by which they can target delivery of substances to the site of brain pathology; and delves into current trends in the application of dendrimers to drug and gene delivery, and diagnostic imaging, in glioma. Finally, future directions for translating these in vitro and in vivo findings to the clinic are discussed.

## 1. Introduction

Gliomas are a common type of primary brain tumor, accounting for about a third of all primary brain tumors and 80% of malignant ones [1]. They are thought to arise from abnormal neuroglial progenitor cells [2,3] and have no cure, making gliomas a leading cause of cancer death worldwide [4]. Age correlates with increased glioma risk; however, gliomas can occur in both children and adults. The incidence of glioma is 6.6 per 100,000 people, and about half are classified as glioblastomas (GBMs). GBM is the most common and aggressive central nervous system (CNS) malignancy, characterized by high inter- and intra-tumor molecular heterogeneity, prominent parenchymal invasion, rapid mutation rate, proximity to eloquent areas of the brain, and the presence of treatment-resistant cancer stem cells [5,6]. Despite decades of intensive research efforts, the prognosis for such aggressive gliomas remains dismal. Indeed, the five-year survival rate for patients with any type of primary malignant brain tumor is about 20% [7].

Rapid advances in molecular genetics have necessitated the creation of new CNS tumor guidelines by the World Health Organization. These guidelines, developed in 2016 and then revised in 2021, use largescale genetic analyses of patient tumor samples to group gliomas into molecular types and subtypes based on their natural histories and treatment responses, thereby reflecting the importance of genetic drivers in tumor progression [8]. As genetic analysis is increasingly being incorporated into patient diagnostic workups, the importance of developing cell signaling modulators, immunotherapies, and gene therapies has become apparent, as has the role of personalized therapy [9].

Even with these advances, the challenges in treating aggressive glioma remain formidable. Current treatment regimens are based on the triad of surgical resection, chemotherapy, and radiotherapy [1]. While total gross resection of glioma is sometimes possible, highly invasive gliomas such as GBM make complete resection difficult and can result in damage to healthy tissue, resulting in post-surgical deficits. Therefore, systemic chemotherapy plays an important role in glioma treatment. However, several factors limit the effectiveness of this approach. Current chemotherapeutic regimens can produce high toxicity with a minimal therapeutic effect. Additionally, frequently used agents, such as temozolomide (TMZ), can result in drug-resistance upon tumor recurrence [10]. Importantly, systemically administered drugs must overcome the blood–brain barrier (BBB), the function of which is to protect the brain by tightly controlling inflow and outflow of substances. Likewise, the blood–brain tumor barrier (BBTB), a region of non-uniform permeability and tissue disruption surrounding the neoplasm, must also be traversed [11]. These barriers often lead to poor penetrance into glioma tissue and lowered treatment efficacy of peripherally administered drugs [12].

A few logical strategies exist to overcome these delivery challenges. One strategy is to bypass the BBB and BBTB by delivering the therapy centrally via intranasal or intrathecal administration, convection-enhanced delivery (CED), or intratumor implants [13,14]. A second strategy is to identify new lipophilic compounds or modify existing drugs so that they can successfully traverse these barriers in their free state using receptor-meditated endocytosis, transcytosis, and transporter uptake. A third strategy is to develop vehicles that can encapsulate or be conjugated to therapeutic cargo for efficient targeting and delivery across anatomic barriers.

This third strategy has been the focus of an explosion of research within the field of nanotechnology. Central to this approach is the use of nanocarriers to efficiently deliver drugs and genomic materials to the brain. Due to their extremely small size, nanomaterials can condense cargo, cross anatomic barriers, and efficiently permeate tumor environments. This latter characteristic can be attributed to the enhanced permeability and retention (EPR) effect, by which disorganized and highly vascular tumors with low levels of lymphatic drainage can be passively targeted [15]. Nanomaterials can also be used for active tumor targeting by attaching ligands corresponding to receptors over-expressed or exclusively expressed on BBB endothelial cells and cancer cells, or by using monoclonal antibodies. The most extensively studied nanomaterials include liposomes and polymeric nanoparticles; however, another class of nanomaterials called dendrimers have more recently been recognized as having distinct characteristics well-suited for targeting substances to brain tumors. Due to their branching structure, dendrimers have a plethora of binding sites, making them multi-functional and highly tunable. They are also versatile with respect to size, can be synthesized from a variety of materials, and have highly modifiable surfaces. This review provides an outlook on the current ways in which dendrimers are designed and applied in the context of glioma and includes a discussion of future directions for translating these promising preclinical findings.

## 2. Properties of Dendrimers

### 2.1. Qualities of Dendrimers

Dendrimers are globular, homogenous, highly symmetric molecules with a regular, highly branched architecture consisting of an interior core surrounded by repeating layers [16,17]. Dendrimers consist of individual units called dendrons, which include the functional core and an attached chemical group [18]. The structure of dendrimers can be highly modified depending on the central atom and functional groups, which in turn affects the functional properties of the dendrimer, allowing significant control over the molecule’s characteristics [16]. In particular, the size, shape, number of generations, branch length, charge, and functional groups can be modified through chemical reactions. Surface charges can be cationic, anionic, or neutral, allowing transport of either hydrophobic or hydrophilic cargo [16]. A plethora of branching molecules can be used to form the structure of the dendrimer, including peptides, glycopeptides, and oligosaccharides [19]. Indeed, their high surface area and high degree of functionality allow them to transport a wide range of therapeutic or imaging agents, rendering them favorable tools for drug delivery, imaging, and theranostics [20].

Divergent and convergent syntheses are used to produce dendrimers; each reaction produces an extra generation. Divergent synthesis begins with the core molecule and grows outwards, whereas convergent synthesis starts with the periphery. Convergent synthesis usually achieves a greater degree of purity compared to divergent synthesis, as divergent synthesis results in multiple reactions being performed on one molecule, whereas convergent synthesis uses a small number of reaction sites during each stage, limiting the number of side products generated [21]. However, the divergent approach is generally a faster and cheaper method that allows for the rapid production of large dendrimers [19].

**Figure 1:**
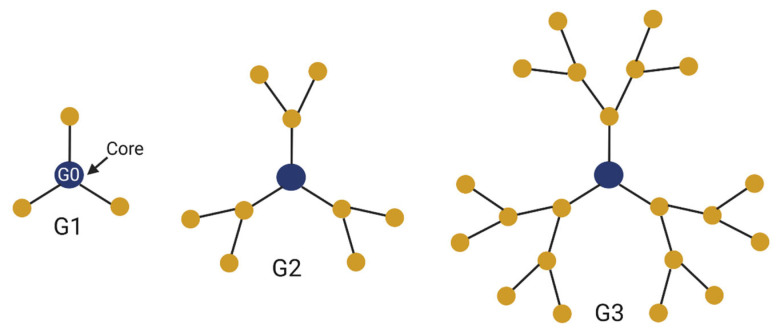
Schematics of dendrimers with one, two, and three generations. Each generation creates new moieties for attachment of functional ligands and therapeutics.

The size of dendrimers is determined by the number of generations. There are reports of dendrimers constructed with as many as thirteen generations while retaining stability across changes in pH and temperature [22]. Importantly, even large dendrimers remain on the nanoscale. A diameter of 30 nanometers was reported for the aforementioned dendrimer consisting of thirteen generations [22]. More generations result in more surface functional groups, allowing for more drug conjugation and delivery (Figure 1). Moreover, multi-functional core molecules can be selected, allowing for a larger number of end groups at the surface layer compared to mono-functional cores [18]. Liaw et al. demonstrated in a mouse glioma model that increasing the number of generations in a dendrimer improves accumulation, specificity, and retention within the tumor. Dendrimers greater than 8 nm in size can avoid the rapid clearance by the kidneys that often occurs with smaller dendrimers [23]. However, rapid clearance helps mitigate systemic toxicity, and larger-generation dendrimers have been shown to exert greater toxicity against healthy neurons compared to smaller dendrimers [24,25].

Dendrimer cytotoxicity is dependent on the number of generations, overall charge, and constituent dendrons. Cationic dendrimers and those with a large number of generations generally exert greater cytotoxicity compared to smaller, anionic, or neutral dendrimers [26]. Although this may be favorable for tumor targeting, the adverse side-effect profile is undesirable. Modification of the surface layer with polyethylene glycol (PEG) or carbohydrates can reduce the cytotoxicity and should be considered during in vivo testing [26,27].

In addition to their multifunctionality and capacity to transport a range of therapeutic agents, dendrimers can also exert anti-inflammatory effects. Neibert et al. showed that the hydroxyl terminal groups of small dendrimers could inhibit the active sites of the enzymes inducible nitric oxide synthase and cyclooxygenase-2, thereby inhibiting prostaglandin synthesis and inflammation [28]. Separately, Dernedde et al. showed that dendritic polyglycerol sulfates could inhibit the cellular adhesion molecules L-selectin and P-selectin, which regulate trafficking of inflammatory white blood cells and also inhibit components of the complement cascade [29]. Additionally, third and fourth-generation phosphorus dendrimers have been shown to inhibit the release of proinflammatory cytokines from macrophages in in vitro and in vivo mouse models [30]. Certainly, the innate anti-inflammatory effects of dendrimers can be enhanced by conjugating anti-inflammatory therapeutic agents to the molecules, and studies have investigated dendrimer-based drug delivery to modulate the inflammatory system for treatment of retinal degeneration, inflammatory arthritis, and other inflammatory pathologies [31,32].

### 2.2. Types of Dendrimers

Dendrimers can be classified according to their constituent core molecules and branching compounds. Several types of dendrimers exist, including polyamidoamine (PAMAM), poly-L-lysine (PLL), and polypropylene imine (PPI) dendrimers [17,33]. Although these dendrimers remain the most thoroughly explored for medical and therapeutic purposes, other categories have been synthesized and described, including polyester, polyether, polyglycerol, citric acid, and triazine dendrimers [34].

PAMAM dendrimers contain ammonia or ethylenediamine at the core and have methyl acrylate and ethylenediamine added to create the branching architecture [35]. These dendrimers were originally synthesized using the divergent approach, although convergent techniques have also been described [36,37]. They have several properties that render them particularly favorable for biological purposes, including favorable biocompatibility and biodegradation profiles, controlled drug release, non-immunogenicity, solubility in water, and functional capacity [35,38]. The hydrophobic core allows for hydrophobic agents to be internalized within the dendrimer, although they can also be conjugated to the surface [39]. Chemotherapeutics and other drugs can be covalently conjugated to the dendrimers, and hydrolysis or cleavage of the linker can be triggered by a certain pH and environmental niche [35]. PAMAM dendrimers, like other dendrimers, are taken up by the reticuloendothelial system, and neutral PAMAM dendrimers can cross the BBB and localize to inflammatory cells and microglia [40].

Lysine-based dendrimers, such as PLLs, also possess a favorable biodegradation and biocompatibility profile, along with many functional amino groups that allow for drug conjugation [41]. These peptide dendrimers are based on a lysine backbone and therefore adopt characteristics of amino acids, allowing for favorable transport in physiological conditions [42]. Modifications, such as coating the surface with PEG, are often required to reduce the density of cationic charges on the surface which can contribute to cytotoxicity [41]. They have intrinsic antiangiogenic activity which promotes tumor cell death, delays tumor growth, and reduces toxicity to healthy tissue [33,43]. PLLs have emerged as a particularly favorable agent for gene therapy, and the cationic charges from the dendrimers and anionic charges from genetic material result in the formation of non-covalent complexes allowing for transport to target tissue [41,42].

PPI dendrimers are a third category of dendrimers commonly investigated for biological purposes. These dendrimers, synthesized using a divergent approach, generally contain 1,4-diaminobutane as the core, although variants can be used, such as an ethylenediamine core [34]. Propylene imine monomers form the branching units of each generation. Their cationic charges can destabilize negatively charged tumor membranes and promote drug penetration [33]. Like PAMAM and PLL dendrimers, they can be modified with functional groups to promote site-specific targeting and delivery of therapeutic agents. PPI dendrimers have exhibited anti-amyloidogenic activity, rendering them of interest in the treatment of Alzheimer’s disease, which is characterized by the formation of β-amyloid plaques and neurofibrillary tangles [44]. They can promote apoptosis and deliver therapeutic agents to tumors, although toxicity to healthy central nervous system tissue can arise from the density of cationic charges, necessitating surface modifications before use in vivo [45,46].

### 2.3. Modifications and Functional Groups

Dendrimers are routinely modified with functional groups that improve tissue targeting, reduce systemic effects, and carry therapeutic agents [20,47]. One common functional change is the addition of PEG to improve drug delivery [48]. PEGylation has a number of beneficial effects on dendrimers, including decreasing immunogenicity, reducing cytotoxicity, improving biocompatibility and hydrophilicity, and increasing the circulation time [20,49]. PEGylated nanoparticles are often considered “stealth” nanoparticles because the PEGylation helps avoid clearance by the reticuloendothelial system and protects the nanoparticles from opsonization and phagocytosis, thereby improving circulation half-life [48,50]. Consequently, PEG is frequently added to dendrimers to improve therapeutic delivery. For example, He et al. showed that PEGylated G4 PAMAM dendrimers encapsulating the chemotherapeutic doxorubicin can inhibit glioma cell growth in vitro and promote accumulation of doxorubicin in tumor tissue [51]. Similarly, Jiang et al. showed that PEGylated PAMAM dendrimers, conjugated with glioma homing peptides that improve uptake by the BBB, are endocytosed by glioma cells and can target the sites of brain tumors [52].

Dendrimers can also be modified to alter the overall charge, thereby minimizing cytotoxicity and altering nanoparticle targeting and binding. For example, acetylation of PAMAM dendrimers can neutralize their cationic charge, thereby reducing the degree of cytotoxicity that results from the interactions of cationic polymers with negatively charged cellular membranes. Moreover, acetylation improves the delivery of genetic material from dendrimers to cells by decreasing interactions between the dendrimer and DNA, allowing for improved intracellular release [53]. Gabrielson et al. showed that acetylation of primary amines in polyethylenimine dendrimers improves delivery of genetic material to cells by improving intracellular unpackaging [54]. However, excessive acetylation counteracts this effect due to reduced polymer buffering capacity. Waite et al. showed that increasing acetylation of dendrimers reduced cytotoxicity but ultimately inhibited therapeutic gene delivery by decreasing endosomal buffering capacity and preventing their escape from the endosomes [55]. Therefore, an optimal degree of acetylation should be considered—one that sufficiently improves unpackaging and reduces cytotoxicity without causing an excessive decline in buffering capacity.

Dendrimers can also be modified with surface-functional ligands that promote binding to cellular receptors and accumulation at target tissue, as will be discussed in later sections.

### 2.4. Therapeutic Attachments

**Figure 2:**
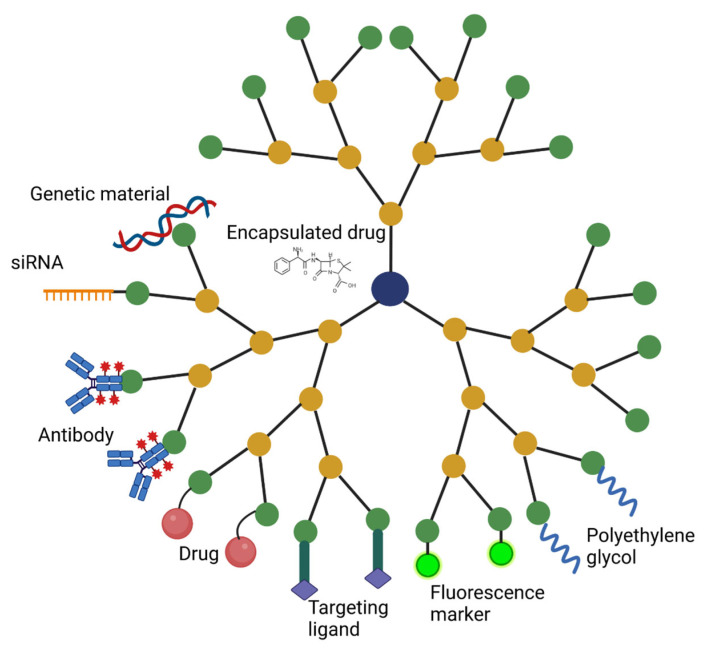
Dendrimer modifications and therapeutic attachments. Drugs can be encapsulated within the inner core or attached to surface functional groups. Dendrimers can carry a range of cargo, including DNA, siRNA, antibodies, and drugs. Addition of polyethylene glycol can improve dendrimer “stealth” and bioavailability. Fluorescence markers can be attached for drug tracking. Ligands can be attached that target specific receptors on the surfaces of cells of interest, improving specificity and minimizing side-effects.

In addition to conjugated agents that improve targeting and reduce systemic side-effects, dendrimers can carry a wide variety of therapeutic agents, either encapsulated within the dendrimer or attached covalently to the surface (Figure 2) [56]. Traditional chemotherapeutic agents often face difficulties in traversing the BBB and penetrating tumor tissue [57,58]. Dendrimers that localize to tumor tissue, which can be achieved by modifying functional groups and attaching targeting agents, as mentioned previously, offer a potential option of improving chemotherapeutic delivery to brain tumors. Hydrophobic agents can be entrapped within the interior cores of some dendrimers, particularly PAMAM and PPI dendrimers; and higher-generation dendrimers offer improved drug solubilization due to their greater internal space available for interactions with drugs [56]. PEGylation can also improve encapsulation via favorable electrostatic interactions and hydrogen bonding, although the efficiency of these interactions decreases when the PEG chains are too large and reduces the volume of the internal space [59]. Additionally, drugs can be attached to the surface via chemical linkages that are cleaved by a tumor microenvironmental trigger, including disulfide, peptide, and ester linkers [56].

Chemotherapeutic agents that have been successfully conjugated to dendrimers include methotrexate, paclitaxel, doxorubicin, cisplatin, and 5-fluorouracil, which have been studied for efficacy against a variety of tumors [56,60,61,62,63]. Conjugation of doxorubicin to dendrimers has been particularly studied for brain tumor treatment. For example, Han et al. showed that PAMAM dendrimers carrying doxorubicin and angiopep-2, which targets the low-density lipoprotein receptor-related protein overexpressed on the BBB and glioma cells, could target tumor tissue and release doxorubicin in a pH-dependent manner [64]. Dhanikula et al. attached methotrexate to glycosylated polyether-copolyester dendrimers, finding that the dendrimers increased the potency of methotrexate in vitro compared to delivery of the free drug alone [65]. Notably, the dendrimers could reduce the size of cells otherwise resistant to methotrexate, demonstrating that the improved potency offered by the dendrimer formulation system may help overcome chemotherapeutic resistance. Furthermore, Lu et al. demonstrated that G5 PAMAM dendrimers could encapsulate arsenic trioxide and promote apoptosis in vitro and in a rodent model of glioma [66]. The chemotherapeutic paclitaxel was also shown to accumulate in the rodent brain after transport via a PPI dendrimer [67].

Beyond the capacity of dendrimers to deliver conventional chemotherapeutics, significant interest has been generated in the use of dendrimers as a delivery vehicle for genetic material, including DNA, RNA, small interfering RNA (siRNA), and microRNA (miRNA) [49]. The flexibility of dendrimers, their spherical and branched architecture, and their polyvalency allow for formation of compact complexes with genetic material [34,68]. These complexes arising from the interaction of DNA with dendrimers have been termed “dendriplexes” [69]. The cationic PAMAM and PPI dendrimers in particular have been explored as nonviral gene delivery vectors, owing to their surface amine groups and positive charges that form favorable ionic interactions with nucleic acids and condense genetic material into small nanoparticles [68]. Modifications of these dendrimers with functional ligands, including amino acids, carbohydrates, polymers, and lipids, can improve cellular uptake and endosomal escape while preventing degradation of the genetic material and minimizing cytotoxicity [68,70].

Endocytosis of the dendriplexes is followed by endosomal destabilization of the complex, resulting in release of the genetic material [34]. DNA can then cross the nuclear membrane and undergo transcription and replication [68]. This strategy has been applied for gene delivery to the brain, as demonstrated by Ke et al., who constructed a PEGylated PAMAM dendrimer conjugated with angiopep and complexed with DNA [71]. Uptake into the endothelial cells of the BBB occurred via clathrin- and caveolae-mediated endocytic processes, and fluorescent markers demonstrated distribution of DNA in vitro throughout the cell and nucleus over time. In vivo fluorescent imaging confirmed accumulation of the DNA in the brains of mice. Significant improvements arose from PEGylation and conjugation of angiopep, and gene expression of GFP was visualized in the cortical and subcortical brain.

Dendrimers can also be used to transport siRNA, which guides the RNA-induced silencing complex to target and cleave complementary RNA [72]. SiRNA can be used for gene knockdown and silencing of genes that promote tumor growth [73]. A similar process can be carried out with miRNAs, which interact with target mRNAs to promote degradation and silencing [74]. However, these RNA molecules are rapidly broken down by enzymes in the systemic circulation, and their negative charge prohibits efficient uptake across cellular membranes [75]. Therefore, conjugation to dendrimers offers an opportunity to improve targeting of siRNA and miRNA to tissues of interest. Indeed, the same features that benefit dendrimers for delivery of DNA apply to the delivery of RNA. For example, Dong et al. designed a dendrimer with a targeting peptide and showed efficient delivery of siRNA to prostate cancer cells [75]. Similarly, Liu reported that PAMAM dendrimers could protect siRNA from enzymatic degradation and improve accumulation of siRNA in prostate cancer cells. The siRNA inhibited a heat-shock protein and in turn produced apoptosis of the tumor cells. Moreover, they showed that the complexes of dendrimers and siRNA had a favorable safety profile [76]. Elsewhere, Taratula et al. combined PPI dendrimers with another type of nanoparticle, superparamagnetic iron oxide nanoparticles, to deliver therapeutic siRNA to tumor cells and improve the efficacy of the chemotherapeutic cisplatin [77]. Their work illustrates how multifunctional delivery systems can be constructed by combining different types of nanoparticles and therapeutic agents.

Other studies have been carried out for the delivery of siRNA and miRNA to brain tissue. Posadas et al. showed that carbosilane dendrimers could deliver siRNA to target translation of the hypoxia-inducible factor-1-alpha protein involved in hypoxic signaling [78]. By knocking down the RNA and preventing protein synthesis, they showed a neuroprotective role for the transcription in chemical hypoxia-mediated neurotoxicity. Kim et al. examined intranasal delivery of a biodegradable PAMAM dendrimer carrying siRNA to adult rats, showing accumulation in the cortex, hypothalamus, amygdala, and striatum 1 h after infusion. The siRNA successfully targeted the RNA for high mobility group box 1, a pro-inflammatory protein, thereby reducing the size of an infarct in an ischemic rat brain model [79]. Dendrimer/siRNA complexes have also shown promising effects for targeting gliomas. Liu et al. illustrated that a PAMAM dendrimer could carry the miR-7 gene to glioma cells to silence the expression of genes involved in the epidermal growth factor receptor (EGFR) pathway, which promotes tumor growth and spread [80,81].

## 3. Delivery and Targeting Mechanisms

### 3.1. Systemic Delivery

The pharmacodynamics, pharmacokinetics, and biodistribution of dendrimers are dependent on their respective sizes, structures, charges, and surface functional groups. Small dendrimers undergo renal filtration. For example, PAMAM dendrimers are filtered through the kidneys up through generation-five compounds, and larger dendrimers cannot be filtered at the renal glomerulus and instead undergo hepatic clearance [82,83,84]. The negatively charged basement membrane of the renal glomerulus can present a barrier to filtration of anionic dendrimers, favoring the hepatic pathway, particularly for those anionic dendrimers close to the filtration size threshold [84]. Clearance by the reticular endothelial system, kidneys, and liver is a critical consideration in the design and evaluation of dendrimers designed to target gliomas.

Dendrimers are usually administered intravenously or subcutaneously, and some studies have characterized an oral route that improves intestinal drug permeability [82]. Their overall charge is a significant determinant of their uptake by target tissue. Cationic dendrimers interact readily with negatively charged cellular membranes, promoting their cellular uptake and rapid clearance from plasma [82]. Neutralization of the surface charge, as can be achieved with acetylation or PEGylation, increases the significance of size and hydrophobicity to dendrimer uptake. Neutralization of surface charges can improve circulation time of dendrimers by reducing the rapid clearance by the renal filtration system [82].

**Figure 3:**
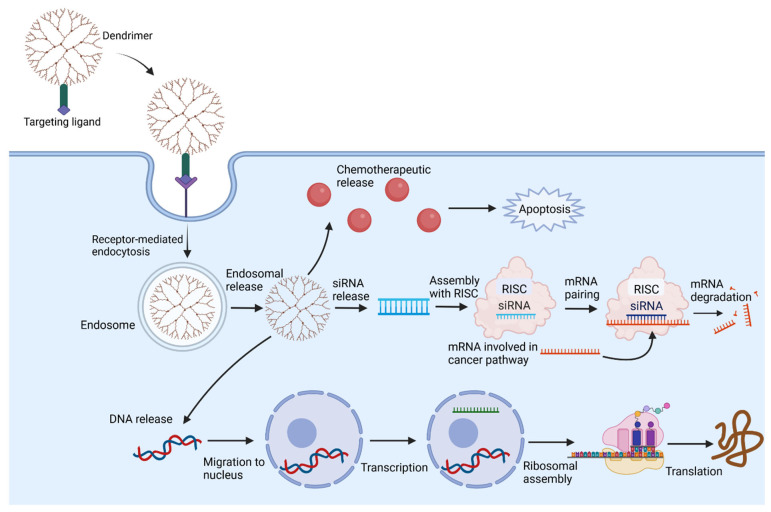
Dendrimers can enter the target tissue via several mechanisms, including receptor-mediated endocytosis, as depicted. Once inside, dendrimers can exert a variety of effects depending on their associated functional groups. Dendrimers can release chemotherapeutic drugs encapsulated within their core or conjugated to their surface to promote apoptosis of tumor cells. Alternative uses of dendrimers include release of genetic material. Release of siRNA can allow for selective degradation and inhibition of mRNA involved in tumor development, proliferation, and survival. Dendriplexes release DNA that travels to the nucleus and undergoes transcription and translation to produce proteins, a novel form of gene therapy.

Surface ligands that function as targeting moieties can improve dendrimer uptake by a selected tissue. These ligands include monoclonal antibodies, carbohydrates, peptides, and small-molecule receptor agonists that bind cellular receptors overexpressed on the target tissue [82,85]. For example, Patri et al. conjugated folic acid to the surfaces of dendrimers to target tumor cells overexpressing the folic acid receptor, allowing for internalization of the dendrimer and release of methotrexate from the dendrimer [86]. This study also illustrates receptor-mediated endocytosis as a mechanism of dendrimer uptake, in which binding of the dendrimer to a receptor triggers recruitment of clathrins and adaptor proteins to the plasma membrane, which bends the membrane and forms a clathrin-coated vesicle [87]. Membrane invagination results in internalization of the vesicle with the dendrimer, and the endocytic protein machinery is uncoated, allowing for intracellular trafficking of the vesicle and fusion with an early endosome (Figure 3) [88]. An alternative pathway is the caveolae-mediated endocytic pathway, governed by the integral proteins of the caveolin family, which similarly act to promote membrane invagination and internalization of dendrimers [89]. Goldberg et al. illustrated that both clathrin- and caveolin-mediated endocytic pathways are responsible for uptake of dendrimers into intestinal Caco-2 cells [90]. Dendrimer charge also influences the mechanism of uptake. Perumal et al. showed that caveolae-mediated endocytosis is primarily responsible for uptake of anionic dendrimers by lung epithelial cells, whereas a separate, non-clathrin, non-caveolae mediated mechanism governs uptake of cationic and neutral dendrimers by these cells [91]. The mechanism of entry is an important consideration when determining the intracellular target of interest.

### 3.2. Systemic Toxicity

The toxicity of dendrimers is an important consideration in their synthesis and testing, and higher-generation dendrimers often confer greater toxicity compared to lower-generation dendrimers [83]. Dendrimers with cationic charges, such as PAMAM and PPI dendrimers, can interact with and destabilize cellular membranes, producing nanoscale holes in the lipid membranes that allow leakage of material and increase membrane permeability [92,93,94]. Pryor et al. illustrated that G6-amine PAMAM dendrimers were significantly more toxic than neutral and anionic PAMAM dendrimers at the same concentration in embryonic zebrafish, illustrating the important influence of surface charges. Toxicity was measured by assessing mortality, developmental progression, and physical malformations [95]. The effect of surface charge on cytotoxicity was also assessed by Greish et al., who compared cationic amine-terminated dendrimers with carboxyl- and hydroxyl-terminated dendrimers. The latter could be administered safely at 50-fold higher doses compared to the cationic dendrimers, which produced a coagulation cascade and were fatal in mice at doses above 10 mg/kg [96]. Chen et al. showed that PEGylated and anionic dendrimers are significantly less cytotoxic and hemolytic compared to cationic dendrimers [97].

Additionally, cationic dendrimers have been associated with a pro-coagulant role and can induce platelet aggregation in vivo, resulting in a coagulation cascade that can produce a syndrome similar to disseminated intravascular coagulation. Jones et al., in a study also using embryonic zebrafish, illustrated that G7 PAMAM dendrimers can interact with a variety of blood components, including platelets, fibrinogen, and anionic blood proteins, producing a unique, multi-component, rapid coagulation cascade. These effects appear dependent on dendrimer size and charge [98]. PAMAM dendrimers can also be endocytosed into lysosomes and increase the lysosomal pH, triggering a lysosomal apoptotic pathway via release of lysosomal enzymes. These cytotoxic effects are most significant for cationic amine-terminated dendrimers. Heavily acetylated dendrimers lack cytotoxic effects due to the neutralizing effect of acetyl groups [94]. Similarly, Kolhatkar et al. illustrated that increasing acetylation of G2 and G4 PAMAM dendrimers can reduce cytotoxicity on Caco-2 epithelial cells by more than 10-fold and decrease nonspecific binding [99].

### 3.3. Targeting Brain Tissue

Targeting of dendrimers to gliomas requires dendrimers to either bypass or traverse the selective BBB, and to accumulate in the desired target tissue. Techniques that avoid the BBB include intracerebral implants, such as biodegradable polymer wafers that can deliver chemotherapeutics, and CED, which uses an external infusion pump to deliver drugs directly into the brain parenchyma via a pressure gradient [100,101]. Intrathecal delivery strategies can also be used, in which the therapeutic is delivered into the cerebrospinal fluid spaces. Dai et al. injected G4 PAMAM dendrimers into the subarachnoid space of rabbits with inflammation-induced cerebral palsy, finding that they accumulated in activated microglia and astrocytes within the periventricular white matter, which are responsible for neuroinflammation, even without targeting moieties [102]. Neelov et al. studied the efficacy of dendrimers as anti-amyloidogenic agents by injecting G3 and G5 polylysine dendrimers into the intraventricular space in rats. They found localization in the cortex and hippocampus within three hours [103]. Additionally, Kim et al. delivered PAMAM dendrimer-conjugated triamcinolone acetonide to the intrathecal space, demonstrating that it could inhibit the activation and expression of microglial pain-related genes in the spinal cord of a mouse model with peripheral nerve injury, thereby reducing neuropathic pain [104].

Several of these drug delivery methods were analyzed by Albertazzi et al., who assessed G4 PAMAM dendrimer diffusion using confocal imaging after intraparenchymal, intraventricular, and subarachnoid injection [105]. All three methods allowed for diffusion of dendrimers throughout the brain; the surface functional groups influenced the extent of diffusion. Dendrimers with extensive hydrophobicity from lipid chains were prevented from rapid diffusion, likely reflecting lipid interactions with cellular membranes and the extracellular matrix. These drug delivery strategies avoid the BBB and reduce systemic toxicity; however, they are invasive techniques that are limited by the area of distribution, and intrathecal delivery is associated with a high degree of turnover from clearance of cerebrospinal fluid [106]. Intranasal delivery is a solution that non-invasively avoids the BBB [107]. Katare et al. administered the antipsychotic medication haloperidol via a dendrimer formulation and found significant distribution in the brain after intranasal injection compared to the intraperitoneal route, allowing for administration of a dose 6.7× less in magnitude via the intranasal route while achieving comparable behavioral responses [108]. Separately, Win-Shwe delivered PAMAM dendrimers via a single intranasal administration to mice and found alteration of genetic expression in neurons 24 h after administration, including upregulation of brain-derived neurotrophic factor signaling pathway molecules in the hippocampus and cerebral cortex [109]. Modifications to the dendrimers can improve intranasal transport: Xie et al. showed that combining G5 PAMAM dendrimers with an in situ-stable gel allowed significant uptake by the brain, reaching peak accumulation at 12 h [110]. However, the intranasal route is limited by the volume of drug that can be sprayed, irritation and damage to the mucosa and cilia from administration, and clearance from the nasal cavity [107,111].

Alternatively, nanoparticles can be delivered into the systemic circulation and cross the endothelial cells lining the BBB via paracellular diffusion, particularly with small nanoparticles, and/or transcellular transport mechanisms, including carrier-mediated transport, adsorptive-mediated transcytosis, and receptor-mediated endocytosis [111,112]. Receptor-mediated endocytosis into the endothelial cells can be facilitated by surface conjugation of functional groups that bind receptors on the BBB, including the insulin, transferrin, and apolipoprotein E receptor [111]. For example, Huang et al. conjugated transferrin to PEGylated PAMAM dendrimers and found improved uptake by brain-capillary endothelial cells compared to dendrimers lacking the transferrin ligand [113]. Moreover, intravenous administration of these dendrimers complexed to DNA resulted in greater upregulation of gene expression in the mouse brain compared to the non-transferrin dendrimers. Somani et al. also showed that intravenous administration of PPI dendrimers carrying DNA and conjugated to transferrin improved uptake of the genetic material and doubled the amount of gene expression in the brain in vivo compared to dendrimers lacking the transferrin ligand [114]. Later, Huang et al. showed even greater uptake by BBB endothelial cells of a PEGylated PAMAM dendrimer conjugated to lactoferrin compared to the same dendrimer conjugated to transferrin, illustrating the significance of the surface functional group for mediating the interactions of the dendrimer with cellular receptors [115]. Similarly, Ke et al. targeted the low-density lipoprotein receptor-related protein-1 on brain capillary endothelial cells using angiopep conjugated to PAMAM dendrimers complexed with DNA in an in vivo mouse study [71]. They found receptor-mediated endocytosis and a caveolae-mediated process responsible for uptake of these dendrimers, resulting in improved diffusion across the BBB and greater efficacy in gene expression compared to unmodified dendrimers.

### 3.4. Targeting Brain Tumors

After dendrimers reach the CNS, the next step is targeting to the brain tumor to minimize side-effects from cytotoxicity to healthy nervous tissue. Nanoparticles can accumulate at the site of brain cancer via the EPR effect, a well-documented phenomenon reflecting the irregular and extensive neovascularization characteristic of fast-growing tumors with high metabolic and oxygen requirements. Permeability is increased along these blood vessels, owing to larger gaps between the endothelial cells, allowing for preferential accumulation of nanoparticles at the tumor site [116]. Expression of inflammatory mediators at the tumor site further promotes the EPR effect, and impaired lymphatic drainage from solid tumors promotes retention of therapeutics [117].

However, the magnitude of the EPR effect can vary among CNS malignancies. Targeting of dendrimers to brain tumors can be improved by conjugating functional ligands that bind overexpressed receptors on the tumor cells [118]. These functional changes are broad in scope, including carbohydrate moieties, PEG, folic acid, biotin, and peptides.

The addition of sugar moieties to dendrimers can improve targeting of tumor tissues by exploiting the Warburg effect, in which cancer cells preference anaerobic glycolysis over oxidative phosphorylation [119]. Cancer cells overexpress glucose transporters and thereby increase uptake of glucose, and nanoparticles are often glycosylated to increase their uptake by tumors [120]. Although limited studies have investigated glycosylation of dendrimers, Sharma et al. showed that attaching carbohydrate moieties, including glucose and galactose, improves transport of dendrimers across glucose transporters in GBM tissue [47]. The various types of sugars resulted in differential effects. Glucose attachment improved uptake by tumor-associated macrophages (TAMs), and galactose interacted with galactins on the surfaces of GBM cells to enhance their uptake by the tumor microenvironment. Conjugation of mannose to G4 PAMAM dendrimers has been shown to globally alter biodistribution and improve uptake via mannose receptor-mediated endocytosis in a neonatal brain injury model while preserving the inherent capacity of the nanoparticles to target neuroinflammation [121].

Folic acid and biotin are vitamins that can enhance targeting of brain tumors by dendrimers. Folic acid plays an essential role in nucleotide synthesis required for the division and proliferation of tumor cells, and consequently many cancer cells, including gliomas, significantly overexpress cysteine-rich folate receptors compared to healthy tissue [122]. Xu et al. therefore conjugated folic acid to G4 PAMAM dendrimers and found that conjugation improves uptake of the nanoparticles by head and neck cancer cells [123]. Separately, conjugation of folic acid to G5 PAMAM dendrimers carrying the chemotherapeutic doxorubicin increased uptake by glioma cells in vitro compared to dendrimers not modified with folic acid. Moreover, the dendrimer conjugates improved median survival of rats implanted with gliomas compared to free doxorubicin alone [124].

Similarly, biotin receptors are overexpressed in cancer cells, given the role of biotin in promoting cellular growth [125]. Furthermore, biotin improves uptake of nanoparticles by the endothelial cells lining the BBB [126]. Consequently, biotinylation offers the possibility of improving BBB uptake and tumor targeting. Uram et al. showed that biotinylated G3 dendrimers carrying celecoxib improved apoptosis of GBM cells in vitro and reduced proliferation compared to administration of the free drug alone [127]. Later, they showed that biotinylation of the dendrimers similarly improved uptake and induced apoptosis in GBM cells resistant to the chemotherapeutic TMZ, which often develops after initial TMZ treatment [128].

Monoclonal antibodies can also improve dendrimer targeting while improving in vivo stability. Antibodies conjugated to dendrimers can target specific receptors on target tissue to promote dendrimer uptake [129]. This strategy was used by Otis et al. to target breast cancer cells overexpressing the human EGFR-2. G5 PAMAM dendrimers were conjugated with trastuzumab, a humanized antibody targeting this receptor, resulting in selective binding and uptake by the cancer cell lines [130]. Similarly, Marcinkowska et al. showed that trastuzumab can improve targeting of PAMAM dendrimers carrying doxorubicin [61]. Wu et al. showed that a boronated PAMAM dendrimer could be targeted towards glioma cells in a rat model by attaching cetuximab, an antibody targeting the EGFR that is overexpressed on tumor cells [131].

## 4. Applications of Dendrimers in Glioma

**Figure 4:**
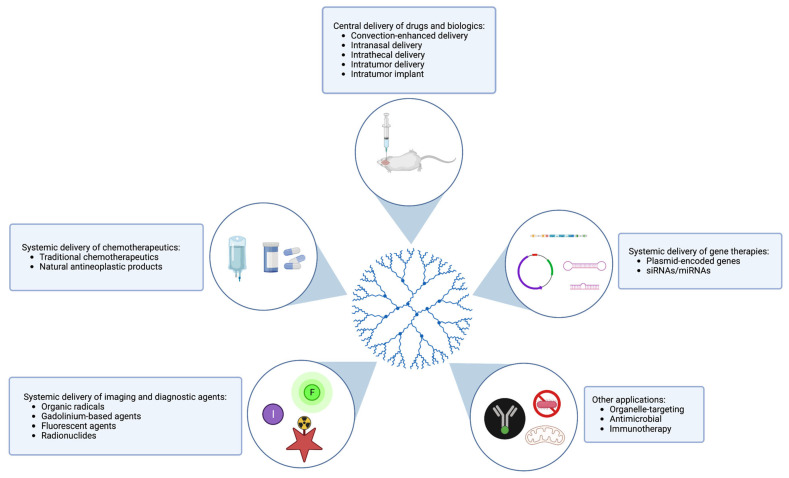
Potential applications of dendrimers in the treatment and diagnosis of glioma. Dendrimers and dendrimer conjugates with functional and targeting moieties attached have been used to deliver chemotherapeutic drugs directly to the tumor site when delivered systemically or intratumorally. Dendrimer conjugates can also be used to deliver biologics, including DNA- and RNA-based agents, to tumor sites for the purposes of gene therapy. In addition, dendrimers can be used for imaging and diagnostic purposes, including as agents for MRI/PET/SPECT modalities. Other novel applications of dendrimers in glioma include organelle-specific targeting, antimicrobial activity, and immunotherapy.

There are a range of ways in which dendrimers are being used in the treatment and diagnosis of glioma (Figure 4). Several novel dendrimer-based delivery, imaging, and other applications are detailed in the sections below.

### 4.1. Delivery of Drugs and Chemotherapy

A logical way in which dendrimers can be applied to the treatment of glioma is as vehicles for delivering drugs from the periphery to the tumor target site (Table 1). This is especially important for drugs that are unstable in physiologic conditions or have high toxicity that prevents them from being used at therapeutic doses. TMZ, a therapeutic widely used for glioma, has served as cargo in several in vivo studies testing such dendrimer-chemotherapeutic conjugates [132,133]. TMZ is a good candidate for vehicle-assisted administration given its systemic toxicity, severe side-effects, and tendency to degrade in the bloodstream. These studies have resulted in an improved TMZ pharmacokinetic profile when co-delivered as a dendrimer conjugate. Combination treatment with TMZ and other chemotherapeutic drugs is also being explored. There is potential for dendrimers to deliver one or both drugs, which may curb acquired TMZ resistance.

Other chemotherapeutics not normally used for the treatment of glioma—either due to poor BBB penetrance or intolerable systemic toxicity—have been conjugated to dendrimers and tested in rodent models. For instance, cisplatin-loaded dendrimers delivered intracranially via CED resulted in a pronounced antineoplastic effect accompanied by a tolerable toxicity profile in rats [134]. Similarly, systemic delivery of doxorubicin-loaded dendrimers with BBB-targeting moieties demonstrated increased tumor accumulation and permeability, increased tumor growth inhibition, and increased median survival time in C6 glioma xenograft mouse models [124,135]. A similar study in a U-87 glioma mouse model found a reduction in cardiotoxicity and weight loss in the doxorubicin-loaded dendrimer group compared to mice treated with free doxorubicin, indicating an improved side-effect profile [136]. Dendrimer-doxorubicin biodegradable intratumor implants have also been designed in an effort to minimize the systemic side-effects of chemotherapy, though future work is necessary to compare these implants to systemic delivery of dendrimer–doxorubicin conjugates [137]. Even when administered systemically, chemotherapeutic–dendrimer conjugates can localize to specific cell types across the BBB, as was shown in a study by Sharma et al. using intravenous administration of rapamycin-conjugated dendrimers to target TAMs in the context of an orthotopic GBM mouse model [138]. Rapamycin-containing TAMs were then able to release drug into the tumor microenvironment.

In addition to traditional chemotherapeutic agents, natural products with antineoplastic activity but nonspecific targeting can be salvaged for glioma therapy by shepherding them to their desired site of action using dendrimer complexes. Such an approach has recently been used to deliver α-mangostin—a xanthone with antineoplastic properties but poor water solubility and low selectivity for cancer cells—to glioma cells using a biotin-transport-targeting, polysaccharide-modified PAMAM G3 dendrimer [139]. This drug was found to be more effective when bound to a dendrimer compared to its free state, demonstrating that the use of dendrimers in glioma therapy may allow for the targeted use of otherwise nonspecific chemotherapeutic drugs. Similarly, arsenic trioxide (ATO)-dendrimer conjugates administered systemically in U87 and C6 glioma mouse models were found to elicit a prolonged ATO half-life, significantly improved antitumor effect, and prolonged median survival time [66,140].

### 4.2. Delivery of Biologics and Gene Therapy

Gene therapy is a promising approach for the treatment of brain diseases given its ability to manipulate defective genes along relevant pathways associated with disease [141]. With the expanding arsenal of biologics for gene therapy, it is no surprise that dendrimers are under investigation for their potential use in shuttling nucleic acid-based cargo to desired CNS locations. This is particularly important for fragile and easily degradable substances, such as RNA and proteins, which can be unstable under physiological conditions. RNA-based structures are highly susceptible to binding by plasma proteins and enzymatic digestion by endonucleases, and low cellular uptake, necessitating the use of substantially increased dosages [142]. Protein drugs can be immunogenic and subject to enzymolysis and are often too large to cross the BBB. These obstacles loom large in the race to translate potential gene therapies to the clinic.

One alternative to the delivery of free nucleic acids is to use a viral vector, such as adeno-associated virus (AAV), as a vehicle. Viral vectors are frequently used in laboratories as transfection agents, given their innate transduction efficiency; however, their clinical utility as delivery agents is more limited. Despite the now-widespread use and appreciable population-level benefits of several viral vector-based vaccines aimed at preventing and attenuating diseases such as COVID-19, this strategy can have the disadvantages of batch-to-batch vector variation, limited carrying capacity, and safety concerns depending on the vector used, such as rare but severe incidents of vaccine-induced thrombosis [143,144,145]. The clinical use of AAVs as delivery agents for gene therapies is also complicated by reports of viral vector genome integration, potentially resulting in insertional mutations and long-term transgene expression [146].

To avoid these potential safety and integration risks, and to decrease batch-to-batch vector variation and improve target specificity, non-viral vectors can be used for gene delivery. While viral vectors remain more efficient in their payload delivery and require less molecular engineering to ensure target internalization [147], non-viral vectors possess several unique advantages to offset their generally lower transfection efficiency. Dendrimers, notably, are non-viral vectors that have simple preparation methods, high load capacities, and can be manufactured at scale [148]. They can also be customized for cell-specific targeting by modulating their size and surface attachments, and they possess minimal immunogenicity [149]. To this end, a variety of dendrimer-biologic conjugates have been developed and tested using in vivo glioma models.

Plasmids encoding genes of interest, such as those capable of inducing apoptosis or stimulating the immune system, can be shuttled into tumor cells using dendrimers. As proof of this principle, Kuang et al. used intravenous injection of T7 peptide-functionalized dendrimers containing plasmid-encoded siRNA to successfully induce luciferase knockdown in a U87MG glioma nude mouse model treated with luciferin [150]. In another study, intratumoral injection of arginine-modified G4 PAMAM dendrimers carrying a plasmid-encoded interferon beta gene—an immune gene known to possess anti-tumor activity—successfully reduced the tumor size in U87MG tumor-bearing mice [151]. Plasmids encoding apoptosis genes apoptin [152] and tumor necrosis factor-related apoptosis-inducing ligand (TRAIL) [153] have also been delivered to mouse glioma models via dendrimers, resulting in accumulation of conjugates in the tumor region, induction of tumor cell apoptosis, and inhibited tumor growth. In the case of the dendrimer-TRAIL treatment group, more prominent apoptosis was observed in the central tumor region than in mice treated with TMZ, indicating enhanced tumor permeation.

In addition to the delivery of genes promoting apoptosis, dendrimers can be used to deliver biologics that elicit specific gene knockdown. In a recent study, dendrimer-siRNA conjugates were synthesized and shown to be capable of simultaneously extending the half-life of siRNA in plasma and significantly reducing GFP expression in vivo using a CX3CR-1GFP mouse orthotopic GL261 GBM model [154]. Employing confocal analysis, this study found that the dendrimer-siRNA conjugate localized to TAMs and released its payload intracellularly, an important characteristic for minimizing off-target effects. Cell signaling pathways relevant to glioma can also be targeted using mRNAs, as was done by Liu et al. using folate-modified PAMAM dendrimers to deliver miRNA-7 to U251 tumor-bearing mice. [80] This miRNA silenced a collection of genes involved in the clinically relevant EGFR pathway, including EGFR, PI3K, and AKT2, leading to an increased rate of apoptosis, suppression of proliferation, and prolonged survival rate.

### 4.3. Imaging and Diagnostics

In addition to therapeutic delivery, dendrimers are also being explored as novel diagnostic and imaging agents given their low side-effect profiles, ability to breach the BBB/BBTB, and propensity to permeate and accumulate in tumor environments. Magnetic resonance imaging (MRI) is a vital noninvasive and nonradiative imaging tool for diagnosing and evaluating glioma [155,156]. The good safety profile of dendrimers is particularly relevant given the toxicity associated with many metal-based imaging agents, such as the widely used paramagnetic gadolinium (Gd)-based contrast agents, which in rare cases can cause lethal conditions such as nephrogenic systemic sclerosis and may not be suitable for patients with certain pre-existing conditions [157].

Dendrimers with organic radicals anchored to their surfaces (radical dendrimers) can be used as alternative T_1_ contrast agents to Gd-based agents, given that they also have paramagnetic properties to produce contrast but are organic species without the potential for toxicity from metal accumulation. In a recent study, an immunocompetent, orthotopic GL261 murine GBM model was used to test ex vivo and in vivo tumor contrast enhancement using a G3 water-soluble radical dendrimer with nitroxyl radicals anchored to its branches [158]. Intravenous administration of this radical dendrimer was found to produce levels of contrast comparable to commercial Gd-based agents at a quarter of the standard dose with no in vivo toxicity detected; no toxicity symptoms or body weight loss were observed in mice monitored for one month post-administration. Additionally, longer retention times within the tumor were observed, allowing for longer imaging acquisition times compared to Gd agents. Longer acquisition times are particularly useful for high-resolution and quantitative MRI scans, such as those used in the clinical trial and research settings.

Dendrimers carrying traditional Gd-based agents can also be synthesized to improve specific targeting to glioma cells and to combine with other imaging techniques. For example, Karki et al. created a dual-mode MRI and near-infrared fluorescence imaging agent by conjugating GdDOTA (a Gd-based agent) and DyeLight680 (a fluorescent dye) to PAMAM dendrimers. Glioma-specific accumulation was observed when these conjugates were systemically administered to a U251 glioma rat model [159,160]. In addition to its potential clinical utility, this dual-imaging method could be used in in vivo research for precisely visualizing tumor localization of delivered substances through initial pre-mortem MRI imaging with confirmatory post-mortem fluorescence imaging of pathological tissues.

The use of dendrimer platforms in other imaging modalities, including radionuclide-based imaging techniques, has also been explored. Single photon emission computed tomography (SPECT) and positron emission tomography (PET) are extremely sensitive diagnostic strategies; however, some radionuclides have issues with specificity of localization. For instance, ^131^I is a radioisotope that possesses many characteristics making it well-suited for radiotherapy, including its long half-life and convenient labeling methods; however, the affinity of free ^131^I for the thyroid is problematic. Zhao et al. circumvented this issue by attaching ^131^I to a chlorotoxin-targeting dendrimer system and demonstrated effective tumor localization on SPECT imaging [161]. This finding was confirmed using fluorescent tracing experiments and lays the groundwork for future studies to investigate the theranostic potential of this platform in radiotherapy. Novel positron emission tomography (PET) dendrimer systems are also being developed. In one study, PET reporting units were attached to dendrimers and the EPR effect was exploited for accumulation of the imaging dendrimer within the tumor [162]. Remarkably, this novel dendrimer system was able to detect tumors characterized by low glucose uptake that were image-refractory when using the clinical PET reference agent, [^18^F]FDG. These results merit further study of the use of dendrimer platforms in glioma imaging.

### 4.4. Other Applications

Other potential applications for dendrimers will undoubtedly continue to be appreciated as the fields of nanotechnology and genetic engineering continue to advance. Imaging agents can be loaded onto dendrimers along with therapeutics to promote targeted theranostic approaches [163]. Multiple biologics and traditional chemotherapeutics can be co-conjugated to dendrimers to accomplish dual functions [164]. Organelle-specific targeting can even be achieved, as demonstrated by Sharma et al. using dendrimers conjugated to the translocator protein ligand DPA in a mouse GBM model, which is a potentially useful mechanism for immunotherapy delivery [165]. Silver-based dendrimers, which possess both intrinsic antimicrobial properties and antineoplastic activity, and are small enough to penetrate bacterial biofilms, are even being leveraged as “nanoantidotes” to serve as adjuvants and to prevent post-chemotherapy opportunistic infections [166].

Immunotherapy, a highly successful treatment option for many other types of aggressive cancer, is being actively pursued as a treatment option for GBM, which is characterized by an immunosuppressive environment [167]. Immunotherapeutic strategies, such as monoclonal antibodies and checkpoint inhibitors, might also benefit from specific targeting by dendrimer platforms. For instance, nanoinhibitors targeting relevant glioma signaling pathways might be effective alternatives to monoclonal antibodies, given their decreased cost and lower immunogenicity. To target the aberrant mesenchymal-epithelial transition factor (MET) activation present in a subset of glioma patients, Wu et al. created a dendrimer-based nanoinhibitor containing a MET-targeting peptide [168]. Intravenous administration of this agent into xenografted mice was found to delay tumor growth and increase survival compared to mice treated with free peptide. Another recent study reported that PAMAM dendrimers with an attached BBB-targeting moiety were loaded with both siLSINCT5, a siRNA targeting a lncRNA correlating with poor glioma prognosis, and aNKG2A, an immune checkpoint inhibitor [164]. When this dendrimer conjugate was delivered to an in vivo glioma model, upregulation of natural killer and T cells were observed within tumor tissue, and the survival time of glioma-bearing mice was increased.

**Table 1:** Recent in vivo studies testing dendrimer applications in rodent models of glioma.

	Application in Glioma	In Vivo Glioma Model	Dendrimer Characteristics	Dendrimer Cargo	Results	Reference
Chemotherapy	Delivery of chemotherapeutic via CED	F98 rat glioma	EGFR-targeting G5 PAMAM dendrimer	Cisplatin	Robust antineoplastic effect; tolerable toxicity profile	[134]
	Delivery of chemotherapeutic via intravenous injection	U-87 mouse tumor model	Poly(2-methacryloyloxyethyl phosphorylcholine) G3 PAMAM dendrimer	Doxorubicin	Enhanced tumor targeting; reduced PAMAM cytotoxicity; reduced side effect profile; reduced tumor growth in mice treated with modified dendrimer compared to free drug	[136]
	Delivery of chemotherapeutic via intravenous injection	C6 glioma xenograft mouse model	iRGD-modified G4 PAMAM dendrimer (glioma cell-targeting)	Doxorubicin	Increased vascular permeability of tumor; decreased vascular density of tumor with average vascular diameter; accumulation in brain tumor	[135]
	Delivery of chemotherapeutic via intravenous injection	C6 glioma xenograft rat model	Folic acid-conjugated, borneol-modified PAMAM G5 dendrimer (glioma cell-targeting; BBB-targeting)	Doxorubicin	Improved doxorubicin accumulation in brain tumor; increased tumor growth inhibition; prolonged median survival time	[124]
	Long-term intratumor release of chemotherapeutic	C6 xenograft mouse model	RGD-modified PEGylated PAMAM within biodegradable intratumor implant	Doxorubicin	Increased prevention of tumor growth compared to free doxorubicin implants	[137]
	Delivery of chemotherapeutic via intravenous injection	Orthotopic GL261 GBM mouse model	Ethylenediamine-core PAMAM G4 dendrimer	Rapamycin	Reduced tumor burden; specifically targeted TAMs; reduced rapamycin renal toxicity	[138]
	Delivery of chemotherapeutic via intravenous injection	U-87 glioma xenograft mouse model	iRGD and TGN co-modified PEGylated G5 PAMAM dendrimer (for BBB targeting)	Arsenic trioxide	Enhanced therapeutic efficacy of ATO; prolonged median survival time	[140]
	Delivery of chemotherapeutic via intravenous injection	Orthotopic C6 glioma mouse model	RGDyC-modified PEGylated G5 PAMAM dendrimers	Arsenic trioxide	Prolonged half-life of ATO; improved antitumor effect	[66]
Gene Therapy	Delivery of plasmid-encoded gene via intratumoral injection	Subcutaneous U87MG xenograft model in nude mice	Arginine-modified G4 PAMAM dendrimer	Plasmid-encoded interferon beta gene	Reduced tumor size; selectively induced apoptosis in tumor cells	[151]
	Delivery of plasmid-encoded gene via intravenous injection	C6 xenograft rat glioma model	Transferrin-modified PAMAM dendrimer (BBB targeting)	Plasmid-encoded TRAIL	Conjugate accumulated in tumor; induced apoptosis throughout tumor region	[153]
	Delivery of plasmid-encoded gene via intratumor injection	U87MG xenograft nude mouse model	Arginine-modified G4 PAMAM dendrimer	Plasmid-encoded apoptin gene	Induced apoptosis; inhibited tumor growth	[152]
	Delivery of siRNA via intravenous injection	Orthotopic U87MG glioma nude mouse model	T7 peptide-functionalized PEGylated dendrimers (BBB and glioma cell targeting)	Plasmid-encoded siRNA targeting luciferase	Induced significant knockdown of luciferase expression in glioma (compared to scramble plasmid)	[150]
	Delivery of miRNA via several routes (intravenous, intraarterial, intratumor)	U251 mouse model	Folate-modified PAMAM dendrimer (tumor cell targeting)	miRNA-7	Increased apoptosis rate; increased suppression of proliferation; prolonged survival rate	[80]
Imaging	Delivery of MRI contrast agents via intravenous administration	Orthotopic GL261 GBM mouse model	PROXYL radical dendrimer	N/A	Contrast levels comparable to commercial Gd-based agents; retained longer in tumor	[158]
	Dual-mode MRI and NIR imaging agent delivered via intravenous administration	Orthotopic U251 glioma nude rat model	G5 PAMAM dendrimer	GdDOTA (Gd-based agent) + DyeLight680 (near-infrared fluorescent dye)	Specifically accumulated at glioma site	[159]
	Targeted tumor SPECT imaging and radiotherapy via intravenous administration	C6 glioma xenograft nude mouse model	Chlorotoxin and HPAO-modified, PEGylated, G5 PAMAMs (glioma cell-targeting)	^131^I radioisotope	Effectively targeted tumor	[161]
	Delivery of PET tracers via intravenous administration	Orthotopic U-87 glioma mouse models	Amine-terminated amphiphilic dendrimer	PET reporting units	Able to detect imaging-refractory low-glucose-uptake tumors; favorable safety and pharmacokinetics profile	[162]
Combination Therapy	Delivery of siRNA and immunotherapeutic via intravenous injection	U87 glioma mouse model	tLyp-1-conjugated PAMAM dendrimer (BBB-targeting)	siLSINCT5 (siRNA) and aNKG2A (checkpoint inhibitor)	Correlated with upregulated CD54+/CD69+ NK and CD4+/CD8+ T cells within tumors; increased survival time of glioma-bearing mice	[164]
Other	Inhibition of mesenchymal-epithelial transition factor (MET) signaling via intravenous injection	U87MG glioma xenograft mouse model	PEGylated G4 PAMAM dendrimer	cMBP peptide	Delayed tumor growth on MRI; increased survival in dendrimer-treated mice	[168]

## 5. Discussion and Future Directions

While there is strong interest in both academia and the pharmaceutical industry to translate dendrimers to the clinic for the treatment of patients with glioma, as of this writing, there are no registered clinical trials evaluating dendrimers in the context of glioma (ClinicalTrials.gov), and there are no approved CNS nanomedicines [12]. Indeed, clinical trials for all brain-directed nanocarriers (mainly liposomes and nanoparticles) make up only ~4% of the total trials involving nanocarriers. Yet, an expanding list of preclinical studies of dendrimers indicates their potential value to glioma therapy and diagnosis. Furthermore, many nanoformulations have already advanced to the clinical setting for the treatment of non-glioma cancers, paving the way for CNS-targeted dendrimer formulations to do the same [169,170].

Before this can happen, several preclinical objectives must be attained. Building upon promising previous work to identify targeting ligands for BBB access and glioma-homing [52,171], dendrimer conjugates must be finely tuned to maximize precise targeting of therapeutics and delivery into cells—such as by the attachment of BBB- and glioma-cell-targeting moieties, or the development of pH-sensitive drug offloading systems—while minimizing off-target effects and immunogenicity. Interactions between dendrimer nanocarriers and their therapeutic cargo must be further characterized to determine any level of interference that may affect dosages and efficacy. While the unique branching structure of dendrimers allows for them to be conjugated to many different agents (including targeting agents) using creative chemical synthesis techniques [172], their generation should remain standardized for the purposes of efficiency and reproducibility.

Next, before dendrimer-based therapies and diagnostics can advance to clinical trials, thorough in vivo studies must be performed to evaluate the biodistribution, toxicity, and efficacy of these interventions. Such studies on dendrimers in glioma are currently lacking. The majority of articles report strictly in vitro results, so there are limited pharmacodynamic assessments [12]. These in vivo follow-up studies must be conducted to evaluate successful delivery of the drug, biologic, or imaging probe of interest, and to evaluate for therapeutic effect. Evidence that a dendrimer nanocarrier can successfully penetrate cells in vitro does not necessarily indicate that it can do so when delivered systemically in a living organism; similarly, evidence that a dendrimer nanocarrier successfully distributes at the site of a glioma does not necessarily mean that it can effectively penetrate the cancer cells or release its cargo without interfering with the mechanism of action of the delivered therapeutic. Use of 3D cerebral organoid models has also shown promise for understanding neuroglial interactions and cellular uptake in response to dendrimer treatment [173].

The design of future in vivo studies of dendrimer conjugates can be strengthened by incorporating systemic delivery of dendrimer agents via intravenous routes to best approximate delivery strategies used in patients and minimize complications, such as infection and BBB disruption. Additionally, such studies should strive to report the fractions of free drug and conjugated drug—versus only reporting the total drug concentration in the blood or brain—in order to extrapolate effects on toxicity and to determine the therapeutic index of the dendrimer nanocarrier [12]. Only when these core pharmacologic characteristics of dendrimer conjugates have been elucidated preclinically will these agents have the chance to be investigated in a clinical trial. Finally, the use of dendrimers with multiple therapeutic components, or in combination with existing therapeutic options, should be explored to combat the intratumor heterogeneity and potential for relapse characteristic of aggressive gliomas.

In summary, encouraging findings at the preclinical level indicate that further translational research efforts investigating the use of dendrimers in glioma contexts are well worth the investment and may even lay the groundwork for a new era of CNS-targeted oncologic interventions.

## 6. Conclusions

Remarkably, as evidenced by a rapidly growing volume of preclinical studies, dendrimer conjugates can be designed to effectively bypass the BBB/BBTB, target glioma cells, and contribute to cell death or specific gene knockdown. The unique branching structure and binding capacity of dendrimers allow them to be exploited for novel, multi-step journeys through the peripheral circulation into the brain parenchyma and into individual tumor cells. However, these findings remain firmly in the preclinical realm, as few such approaches have advanced to clinical trials. Dendrimers hold promise to be effective therapeutic delivery and imaging tools for the treatment and diagnosis of glioma, but only if these preclinical results can be effectively translated. Building upon solid and reproducible methodologies for dendrimer conjugate synthesis, future work must focus on validating in vitro results using well-controlled experiments with in vivo tumor models. Additionally, studies investigating the effects of dendrimers and dendrimer conjugates on unloaded cargo must be carried out to demonstrate not only effective targeting, but non-interference with the therapeutic effects. Finally, studies comparing the current standard of care with combination treatments involving dendrimer complexes must be conducted to determine the most appropriate ways in which to incorporate this technology into the care of glioma patients.
